# Inactivated *Lactiplantibacillus plantarum* Ps-8 enhances growth performance and intestinal health in broiler chickens via gut microbiota and serum metabolite modulation

**DOI:** 10.1016/j.psj.2025.105611

**Published:** 2025-07-26

**Authors:** Yangbo Jiao, Weiqiang Huang, Qihang Zhang, Lin Liu, Jie Zhao, Yongfu Chen

**Affiliations:** a Key Laboratory of Dairy Biotechnology and Engineering, Ministry of Education, Inner Mongolia Agricultural University, Hohhot 010018, China; b Key Laboratory of Dairy Products Processing, Ministry of Agriculture and Rural Affairs, Inner Mongolia Agricultural University, Hohhot 010018, China; c Inner Mongolia Key Laboratory of Dairy Biotechnology and Engineering, Inner Mongolia Agricultural University, Hohhot 010018, China; d Inner Mongolia SCITOP Microecological Technology Development Co., Ltd., Hohhot 010018, China

**Keywords:** Inactivated *Lactiplantibacillus plantarum* Ps-8, Broiler chicken, Gut microbiota, Growth performance, Serum metabolite

## Abstract

Inactivated probiotics have gained attention for their positive health impacts, notably enhanced safety, stability, and tolerance, yet their mechanisms remain unclear. In this study, we evaluated the effects of dietary supplementation with inactivated *Lactiplantibacillus plantarum* Ps-8 (ILPs-8) on the intestinal health and growth performance of broiler chickens. A total of 220 one-day-old male 817 broiler chickens (initial weight: 20.97 ± 3.10 g) were randomly allocated into two groups: a control (Con) group receiving a basal diet and an ILPs-8 group receiving a basal diet supplemented with ILPs-8 at a concentration of 500 mg/kg. Supplementation with ILPs-8 significantly improved the growth performance of broiler chickens, especially the average daily gain and feed conversion ratio during the early growth phase (days 1-21) (*P* < 0.05). Furthermore, ILPs-8 markedly enhanced the activities of digestive enzymes and improved the villus morphology in the small intestine (*P* < 0.05). Serum IgG and intestinal sIgA levels were higher in the ILPs-8 group compared to the Con group (*P* < 0.05). Moreover, significant differences in gut microbial composition and functional profiles were observed between the two groups. Interestingly, ILPs-8 supplementation notably increased serum metabolite levels associated with energy metabolism, immune modulation, and anti-inflammatory responses (e.g. l-Glutamine, 2-Hydroxycinnamic acid, 2-Ketobutyric acid, and Spermine). Correlation analyses further indicated potential linkages between growth and immune performance, altered gut microbiota, and serum metabolite levels. Our findings demonstrate that dietary supplementation with ILPs-8 significantly enhanced broiler chicken growth performance and intestinal health. The beneficial effects of ILPs-8 appear to be mediated by modulation of immune responses, gut microbiota composition, and serum metabolite profiles.

## Introduction

According to the Food and Agriculture Organization (FAO), poultry meat is the leading source of animal protein worldwide. China currently ranks as the world’s third-largest broiler producer, generating approximately 14.3 million tons annually, and is the second-largest consumer worldwide (Scanes, 2016). Consequently, the rapid expansion of the poultry industry is essential to satisfy the increasing nutritional demands of the population in the future. Intensive poultry production systems typically involve high stocking densities and enclosed environments, conditions under which birds become highly susceptible to stressors and pathogenic infections, posing significant obstacles to sustainable industry growth (Humam et al., 2020; Nguyen, et al., 2024). Over the past few decades, antibiotics have been extensively used to protect poultry health. However, the lack of stringent regulatory frameworks governing medication use has facilitated antibiotic misuse, creating substantial risks to animal health, food safety and ecological integrity. Antibiotic residues remain in poultry products and waste, adversely impacting animal production and environmental sustainability, and potentially endangering human health through the food chain. Given these concerns, there is an urgent need within the poultry industry to ensure product quality by adopting safer, environmentally sustainable alternatives and antibiotic-free feeding strategies. Therefore, the identification and development of non-toxic, highly efficient, and resistance-free green feed additives has become a central research priority.

Recent evidence suggests that both living microorganisms and non-viable microbial components confer significant health benefits to the host (Ivashkin et al., 2024; Salminen, et al., 2021). Nonliving bacteria exert beneficial effects through metabolites, signaling molecules, and structural components that remain biologically active even after cell death. Compared to live probiotics, inactivated bacteria offer enhanced safety profiles and simpler storage requirements, increasing their suitability for widespread use (Nataraj, et al., 2020; Teame, et al., 2020). Consequently, interest in using inactivated bacterial preparations to improve human and animal health has increased markedly. Emerging studies have demonstrated the potential of inactivated bacteria to improve health. For instance, both heat-inactivated and live *Limosilactobacillus reuteri* GMNL-263 strains effectively alleviated metabolic dysfunction in high-fat diet-induced obese rats by reducing insulin resistance and hepatic steatosis (Hsieh, et al., 2016). Additionally, heat-inactivated *Lactiplantibacillus plantarum* Zhang-LL exhibited superior efficacy compared to its viable counterpart in mitigating chronic ulcerative colitis symptoms in rats, enhancing intestinal microbiota diversity, and significantly improving the microbial community structure (Jin, et al., 2020). While accumulating evidence indicates the beneficial modulation of the gut microbiota by inactivated bacteria, the underlying mechanisms of their interactions with the gut microbiota remain poorly characterized and warrant further investigation.

*Lactiplantibacillus plantarum* Ps-8, isolated from naturally fermented cow’s milk, is recognized as a probiotic strain with substantial potential for application in poultry production (Wang, et al., 2021). Recent research has indicated that *Lactiplantibacillus plantarum* Ps-8 can serve as an effective alternative to antibiotics in broiler production because of its capacity to modulate gut microbiota composition and function. Specifically, this strain accelerates gut microbiome maturation, stimulates protective immune responses, enhances overall immunity, minimizes antibiotic residues, and significantly improves growth performance in broilers (Gao, et al., 2017a, 2017b). Furthermore, *Lactiplantibacillus plantarum* Ps-8 supplementation has been shown to improve growth performance and antioxidant capabilities in oxidatively stressed broilers by positively influencing the cecal microbiota and its metabolites (Zhao, et al., 2023). Additionally, this probiotic has been shown to alleviate immune stress induced by dexamethasone treatment in poultry (Liu, et al., 2021). Although the existing literature highlights the beneficial roles of *Lactiplantibacillus plantarum* Ps-8 in broiler production, limited research has been conducted on the effects of its inactivated form.

In this study, we hypothesized that inactivated *Lactiplantibacillus plantarum* Ps-8 (ILPs-8) would enhance broiler growth performance and immune function by modulating gut microbiota and serum metabolites. Consequently, the primary aim of this study was to evaluate the health-promoting effects, specifically the growth performance and immune function, of dietary ILPs-8 addition to the basal diet of broilers. The secondary objective was to elucidate the underlying mechanisms by examining the alterations in gut microbiota composition and serum metabolic biomarkers induced by ILPs-8 administration. This investigation provides essential scientific insights and practical recommendations for the utilization of ILPs-8 in intensive poultry production, thereby improving broiler health, performance, and production efficiency, and contributes to a broader understanding of the applications of inactivated probiotics in animal husbandry.

## Materials and methods

### Ethics statement

All animal procedures involving animal handling and care were approved by the Institutional Animal Care and Use Committee of Inner Mongolia Agricultural University (approval No: NND2023107).

### Animals and experimental design

**Fig. 1:**
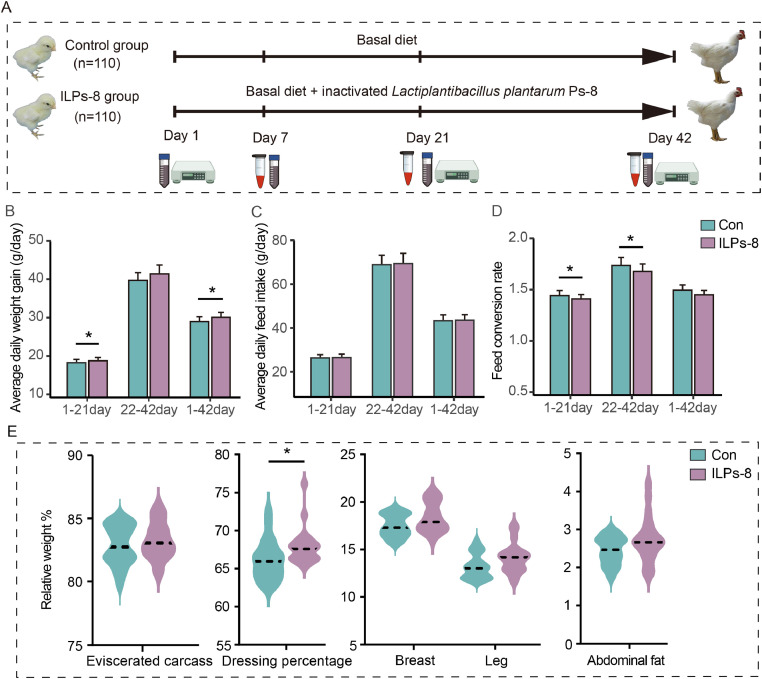
Experimental design and effects of ILPs-8 on broiler growth performance and carcass characteristics (*n* = 10). (A) Interventions, data, and sample collection from the start to the end of the trial. Comparison of (B) average daily weight, (C) average daily feed intake, (D) feed conversion ratio, and (E) carcass characteristics of broilers between ILPs-8 and Con groups. Data were presented as mean ± SEM. Significance was marked by * *P* < 0.05.

A total of 220 one-day-old male 817 broiler chicks (a hybrid of Arbor Acres broiler and Hyline brown), with uniform initial body weights (20.97 ± 3.10 g), were randomly allocated to two treatment groups. Each group comprised 10 replicates, with 11 broilers per replicate. The dietary treatments consisted of a basal diet (control (Con) group) and a basal diet supplemented with ILPs-8 bacterial powder at a concentration of 500 mg/kg (1.0 × 10^10^ cells; designated as the ILPs-8 group). The feeding trial was conducted over 42 days. A schematic representation of the experimental design used to investigate the effects of ILPs-8 supplementation is shown in Fig. 1A. All broilers were housed in standardized cages (dimensions: 88 cm × 84 cm × 47 cm) under controlled environmental conditions with ambient temperature maintained at 24 ± 2 °C, relative humidity between 45 % and 60 %, and a 16-hour photoperiod daily. Feed and water were provided ad libitum during the experimental period. Both treatment groups were managed identically, except for dietary supplementation. The basal diet was formulated according to the broiler nutrient specifications outlined by the Nutrients Requirements of Poultry (National Research Council, 1994). The detailed composition and nutrient profiles of the basal diet and ILPs-8 powder are provided in Table 1 and Tables S1, respectively.

**Table 1:** Composition and calculated nutrient contents of the basal diet.

Items	Diet	
	1–21 d	22–42 d
Ingredients		
Corn	57.00	61.90
Soybean meal (44.2 %, crude protein)	31.30	25.60
Corn gluten meal (60 %, crude protein)	3.90	4.30
Soybean oil	3.10	3.80
Dicalcium phosphate	1.80	1.60
Limestone	1.30	1.20
L-lysine	0.15	0.20
DL-methionine	0.15	0.10
Premix 1	1.00	1.00
Sodium chloride	0.30	0.30
Total	100.00	100.00
Calculation of nutrients		
Metabolizable energy, MJ/kg	12.69	13.10
Crude protein	21.52	19.71
Lysine	1.14	1.04
Methionine	0.50	0.43
Calcium	1.00	0.90
Available phosphorus	0.46	0.42
Arginine	1.36	1.19
Methionine+Cystine	0.85	0.76

1 provided per kg of diet: vitamin A (transretinyl acetate), 10,000 IU; vitamin D3 (cholecalciferol), 3,000 IU; vitamin E (all-rac-α-tocopherol acetate), 30 IU; menadione, 1.3 mg; thiamin, 2.2 mg; riboflavin, 8 mg; nicotinamide, 40 mg; choline chloride, 600 mg; calcium pantothenate, 10 mg; pyridoxine•HCl, 4 mg; biotin, 0.04 mg; folic acid, 1 mg; vitamin B12 (cobalamin), 0.013 mg; Fe (from ferrous sulfate), 80 mg; Cu (from copper sulfate), 8 mg; Mn (from manganese sulfate), 110 mg; Zn (from zinc oxide), 65 mg; I (from calcium iodate), 1.1 mg; and Se (from sodium selenite), 0.3 mg.

### Determination of growth performance and carcass characteristics

The body weights of the broilers were recorded by replicate at the beginning of the experiment (day 1) and subsequently on days 21 and 42, following a 12-hour fasting period. Feed intake was monitored daily per cage. Growth performance parameters, including average daily gain (ADG), average daily feed intake (ADFI), and feed conversion ratio (FCR), were calculated as follows:

$$\text{ADG}=\frac{\text{total}\;\text{body}\;\text{gain}\;\text{of}\;\text{each}\;\text{replicate}\;(g)}{\text{total}\;\text{number}\;\text{of}\;\text{broilers}\;\text{in}\;\text{each}\;\text{replicate}\;\times \;\text{test}\;\text{days}\;(d)}$$

$$\text{ADFI}=\frac{\text{total}\;\text{feed}\;\text{intake}\;\text{of}\;\text{each}\;\text{replicate}\;(g)}{\text{total}\;\text{number}\;\text{of}\;\text{broilers}\;\text{in}\;\text{each}\;\text{replicate}\;\times \;\text{test}\;\text{days}\;(d)}$$

$$\text{FCR}=\frac{\text{ADFI}}{\text{ADG}}$$

On day 42, 10 broilers (one from each replicate) per group, closely matching the group’s average body weight, were selected for slaughter following a 12-hour fasting period to determine their final body weight. The carcass characteristics evaluated included the relative weights of the eviscerated carcass, breast muscle, legs, and abdominal fat. The slaughter yield was calculated using the following equation: eviscerated carcass weight × 100 / broiler live weight (Mahmoudi, et al., 2022). Similar calculations were used to determine the percentages of carcass, breast, leg, and abdominal fat.

### Sample collection

On days 1, 7, 21, and 42 of the experiment, 10 broilers (one per replicate) with body weights approximating the group average were selected and sacrificed by CO_2_ gas (treatment concentration of 45 %) (Ye, et al., 2025). Immediately following euthanasia, samples from the small intestine (duodenum, jejunum, and ileum), cecum and its contents, and the serum were harvested. Briefly, approximately 6-7 mL of blood per broiler was collected and centrifuged at 3,500 rpm for 10 min to separate the serum. Cecal samples and their contents were aseptically collected in sterile, enzyme-free tubes. Small intestine segments were thoroughly rinsed with phosphate-buffered saline (PBS) and subsequently fixed in 4 % paraformaldehyde solution. Serum and cecal samples were immediately frozen in liquid nitrogen, transported to the laboratory, and stored at −80 °C until further analysis.

### Determination of the intestinal histomorphology

Intestinal segments (duodenum, jejunum, and ileum) previously fixed in 4 % paraformaldehyde were dehydrated, embedded in paraffin wax, and sectioned (thickness, 5 μm) using a microtome. Tissue sections were stained with hematoxylin and eosin (HE) following standard procedures for histological evaluation under a microscope (Suvarna, et al., 2013). Morphometric analyses of intestinal histology were conducted according to established protocols using a light microscope equipped with a computer-assisted morphometric analysis system (Li, et al., 2015). Specifically, Leica Qwin V3 software (Nikon Corporation, Tokyo, Japan) was used for image acquisition and analysis to measure the villus height (VH) and crypt depth (CD) for each intestinal segment. Subsequently, the villus height-to-crypt depth ratio (VCR) was calculated as described previously (Alagbe, et al., 2023).

### Determination of digestive enzyme activity

The activities of the digestive enzymes (lipase, protease, and α-amylase) were assessed in the small intestine of individual broilers (one broiler per replicate). Enzyme activity was measured as described by Yaqoob et al. (Yaqoob, et al., 2022). Briefly, the small intestine and its contents were collected, transferred into sterile centrifuge tubes, and diluted 10-fold with PBS. The samples were then centrifuged at 3,000 rpm for 15 min at 4 °C. The supernatants were collected and stored at −80 °C for subsequent analysis. The concentrations of lipase, α-amylase, and protease in the intestinal samples were quantified using commercial enzyme-linked immunosorbent assay kits (Wuhan Xingidi Biological Technology Co. Ltd., Wuhan, China) according to the manufacturer instructions.

### Determination of nutrient digestibility

Nutritional digestibility was determined using the external marker technique, as described in previous studies (Sales and Janssens, 2003). The external marker Celite (1 %) was incorporated into the diet for the final five days before the sampling. Cecal contents were collected at slaughter, pooled by treatment group, and stored at −20 °C until subsequent analysis. Proximate analysis of the feed and cecal contents, including dry matter (DM), curde protein (CP), ether extract (EE), and crude fiber (CF), was performed according to the standardized AOAC procedures (AOAC, 2005). The digestibility coefficients were calculated using the following equation:

$$=100-\left(100\;\times \frac{\text{Marker}\;\text{in}\;\text{feed}\;(\%)}{\text{Marker}\;\text{in}\;\text{ileal}\;\text{contents}\;(\%)}\times \frac{\text{Nutrient}\;\text{in}\;\text{ileal}\;\text{contents}\;(\%)}{\text{Nurtrient}\;\text{in}\;\text{feed}\;(\%)}\right)$$

### Determination of serum and small intestinal levels of immune markers and immune organ index

Small intestinal samples (approximately 10 mL, including intestinal contents) were homogenized with an equal volume of PBS (pH 7.14) and centrifuged at 3,500 rpm for 15 min. Supernatants were stored at −20 °C for subsequent quantification of secretory immunoglobulin A (sIgA). Serum samples were collected on days 21 and 42 of the experiment for immunoglobulin G (IgG) analysis. The concentrations of intestinal sIgA and serum IgG were determined using ELISA with specific chicken sIgA and IgG detection kits (Wuhan Xinqidi Biological Technology, Wuhan, China) according to the manufacturer protocol.

On experimental days 21 and 42, after euthanasia, the immune-related organs (thymus, bursa, and spleen) were carefully excised from each broiler, weighed, and recorded. Immune organ indices (thymus, bursa and spleen indices) were calculated as described previously (Zhang, et al., 2023).

$$\text{Immune}\;\text{organ}\;\text{index}=\frac{\text{organ}\;\text{weight}\;(g)}{\text{body}\;\text{weight}\;(\text{kg})}\times \;100$$

### Metagenomic sequencing and analysis

Metagenomic DNA was isolated from 68 cecal content samples using the QIAamp Fast DNA Stool Mini Kit (Qiagen, Hilden, Germany) according to the manufacturer's instructions. DNA libraries were prepared with an average insert size of approximately 150 bp using Illumina’s NEBNext® Ultra™ DNA Library Preparation Kit. High-quality DNA samples with a concentration greater than 20 ng/μL and purity (*OD*_260/280_) between 1.8 and 2.0 were subjected to paired-end sequencing using an Illumina NovaSeq 6000 platform. Overall, 68 DNA samples were double-ended sequenced and machine-analyzed (Tianjin Novogene Technology Co., Ltd., Tianjin, China). The metagenome data that support the findings of this study have been deposited into CNGBdb database with accession number CNP0006918 (https://db.cngb.org/search/project/CNP0006918/).

The quality control of the generated raw metagenomic sequences was conducted using KneadData software (http://huttenhower.harvard.edu/kneaddata; v0.7.5) employing Trimmomatic remove low-quality reads that were shorter than 60 nucleotides (Bolger, et al., 2014). Host-derived sequences were subsequently filtered by aligning the cleaned reads to the reference genome of broiler chickens using Bowtie 2 software (v2.3.5.1) (Langmead and Salzberg, 2012) with default parameters, thereby ensuring that the final dataset contained only microbial DNA sequences that were suitable for downstream analysis. Functional annotation and microbiome analyses of the curated sequence dataset were performed using the HUMAnN2 pipeline (Franzosa, et al., 2018), a software tool designed for the analysis of microbiome data. Taxonomic profiling at the species level was performed using the MetaPhAn3 software (https://huttenhower.sph.harvard.edu/metaphlan/).

### Determination of short-chain fatty acids (SCFAs) in cecal content

Approximately 0.1 g of thawed cecal contents was transferred into sterile 1.5 mL microcentrifuge tubes, homogenized with 500 μL of distilled water and glass beads for 1 min, and centrifuged for 10 min at 12,000 rpm and 4 °C. A 200 μL aliquot of the supernatant was subsequently transferred to a fresh centrifuge tube, to which 100 μL of 15 % phosphoric acid, 20 μL of internal standard (4-methylglutaric acid), and 280 μL of ether were added. The mixture was homogenized again for 1 min and centrifuged at 12,000 rpm for 10 min at 4 °C. The resulting supernatant was carefully transferred to an injection vial. SCFA concentrations (acetic, propionic, and butyric acids) in the cecal content extracts were quantified using a GC-MS system (TRACE 1300 series GC system, Thermo Fisher Scientific Inc., Waltham, MA, USA) fitted with a capillary column Agilent HP-INNOWAX (30 *m* × 0.25 mm, 0.25 μm). Mass spectrometric detection of metabolites was performed using a Thermo Scientific™ ISQ™ 7000 GC-MS system. The single-ion monitoring mode was used with an electron energy of 70 eV.

### Determination of serum metabolites by liquid chromatography-mass spectrometry (LC-MS)

Serum metabolites were extracted following the protocol described by Demurtas et al. (Demurtas, et al., 2021), with minor modifications. Briefly, serum samples were thawed at 4 °C and vortexed for 1 min. A total of 200 µL of each serum sample was transferred into a 2 mL centrifuge tube, followed by the addition of 400 µL of methanol solution, and vortexed again for 1 min. Samples were centrifuged at 12,000 rpm for 10 min at 4 °C, and the resulting supernatant was carefully aspirated, transferred into a fresh 2 mL centrifuge tube, and evaporated to dryness under vacuum. The dried residue was reconstituted in 150 µL of 2‑chloro-L-phenylalanine solution, vortex-mixed briefly, and filtered through a 0.22 µm membrane. The resulting filtrate was placed in sample vials for subsequent LC-MS analysis. Metabolite profiling was performed using a Vanquish UHPLC system (Thermo Fisher Scientific, USA) equipped with an ACQUITY HSS T3 column (2.1 × 100 mm i.d., 1.8 µm; Waters, USA) and an Orbitrap Exploris 120 mass detector. Quality control (QC) samples were prepared by pooling 10 µL aliquots from each individual sample and analyzed five times to assess the reproducibility and stability of the analytical procedure. Orthogonal partial least squares discriminant analysis (OPLS-DA) was used for dimensionality reduction and classification of the metabolomic datasets. Differential metabolites between groups were identified using univariate statistical analysis (t-test) in combination with multivariate OPLS-DA, applying a cut-off criterion of variable importance in projection (VIP) values greater than 2 and *P-*values less than 0.05 (Zhang, et al., 2022). Metabolite identification was conducted using multiple established databases, including the Human Metabolome Database (HMDB) (Wishart, et al., 2007), LipidMaps (Sud, et al., 2007), the Kyoto Encyclopedia of Genes and Genomes (KEGG) (Wishart, et al., 2007), and massbank (Horai, et al., 2010).

### Statistical analysis

All statistical analyses and graphical visualizations were performed using the R software (v4.1.0) and Adobe Illustrator (v2021). Microbial community structure was analyzed using the vegan package (v2.5.6) to determine alpha diversity and the ggpubr package to perform principal coordinates analysis (PCoA) based on Bray-Curtis distances. Owing to the skewed and non-normally distributed nature of the dataset, differences between groups were statistically evaluated using the non-parametric Mann-Whitney U test. Data are reported as mean ± standard error of the mean (SEM), and statistical significance was defined at *P* < 0.05. Spearman’s correlation coefficient was applied to investigate correlations between differential microbial species, serum metabolites, and phenotypic parameters (cut-off: *r* > 0.4 or < −0.4) (Ma, et al., 2023).

## Results

### Growth performance and carcass characteristics

The growth performance of the broilers was assessed by evaluating their ADG, ADFI, and FCR. During days 1-21, no significant differences in ADFI were detected between the experimental groups (*P* > 0.05). However, the ILPs-8 supplemented group exhibited significantly higher ADG than the Con group (*P* < 0.05), along with a notably improved (lower) FCR (*P* < 0.05) (Fig. 1B-D). From days 22 to 42, FCR was significantly lower in the ILPs-8 group than in the Con group (*P* < 0.05), whereas no significant differences were observed in ADFI or FCR between the groups during this period (*P* > 0.05). Throughout the entire period from days 1 to 42, ADG was significantly higher in the ILPs-8 group than in the Con group (*P* < 0.05), whereas no significant differences were observed in ADFI and FCR (*P* > 0.05). Collectively, these findings indicate that dietary supplementation with ILPs-8 improved growth performance by enhancing body weight gain during the early (1-21 days) and overall periods (1-42 days), along with improved feed efficiency from 1 to 21 days and from 22 to 42 days of age.

Carcass characteristics are reliable indicators of meat quality and economic viability in poultry production systems. Supplementation with ILPs-8 significantly increased the dressing percentage by 1.81 % compared to the Con group (*P* < 0.05) (Fig. 1E), underscoring the potential economic benefits of ILPs-8 supplementation in broiler diets.

### Intestinal histomorphology, digestive enzyme activity and nutrient digestibility

**Fig. 2:**
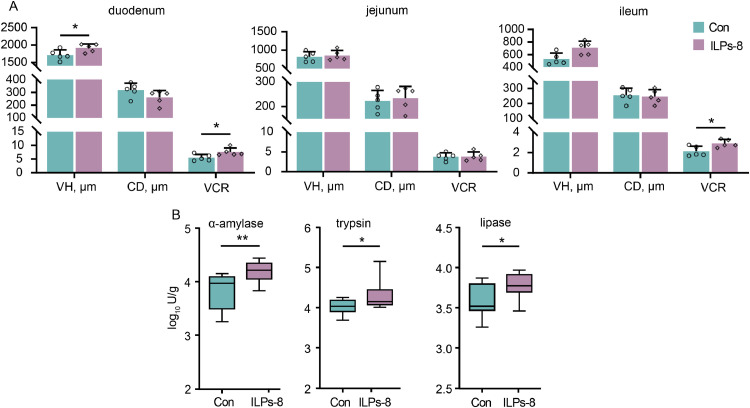
Effect of ILPs-8 supplementation in basic diet on intestinal morphology and digestive enzymes in broilers (*n* = 10). (A) Villus height, crypt depth, and villus height-to-crypt depth ratio in the duodenum, jejunum, and ileum at 42 days. (B) Digestive enzyme activity in the small intestine at 42 days. Significance was marked by * *P<* 0.05, ** *P<* 0.01.

Histological analysis of the small intestine revealed a significant increase in VH in the duodenum and VCR in both the duodenum and ileum following ILPs-8 supplementation (*P* < 0.05). However, no significant alterations were observed in other aspects of small intestinal mucosal morphology (*P* > 0.05) (Fig. 2A). Digestive enzyme activity was assessed in the small intestine on day 42 of the experiment. Lipase, trypsin, and α-amylase activities were significantly elevated in the ILPs-8 supplemented group compared to the Con group (*P* < 0.05). Although ILPs-8 supplementation did not significantly alter the digestibility of DM, EE, and GE in cecal contents (*P* > 0.05), it significantly improved the digestibility of CP and CF by 5.01 % and 1.82 %, respectively (*P* < 0.05) (Table 2).

**Table 2:** Effect of ILPs-8 on the nutrient digestibility of broiler cecum contents on day 42 1.

Items	Diet 2		*P*-Value
	Con	ILPs-8	
Dry matter, %	69.21 ± 1.79	72.57 ± 1.54	0.69
Crude protein, %	62.78 ± 1.13	66.29 ± 0.98	0.016
Gross energy, %	73.72 ± 1.54	75.47 ± 1.38	0.037
Ether extract, %	72.94 ± 3.12	75.12 ± 1.40	0.84
Crude fiber, %	15.37 ± 0.87	17.99 ± 0.39	0.034

1 Values are means of 10 samples per treatment, with their respective standard errors (SEM).

2 Con: a basal control diet without supplementation, ILPs-8: the same basal diet supplemented with 500 mg of inactivated *Lactiplantibacillus plantarum* Ps-8 /kg of feed from day 1 to day 42.

### Relative weight of immune organs and immunological indicators

**Fig. 3:**
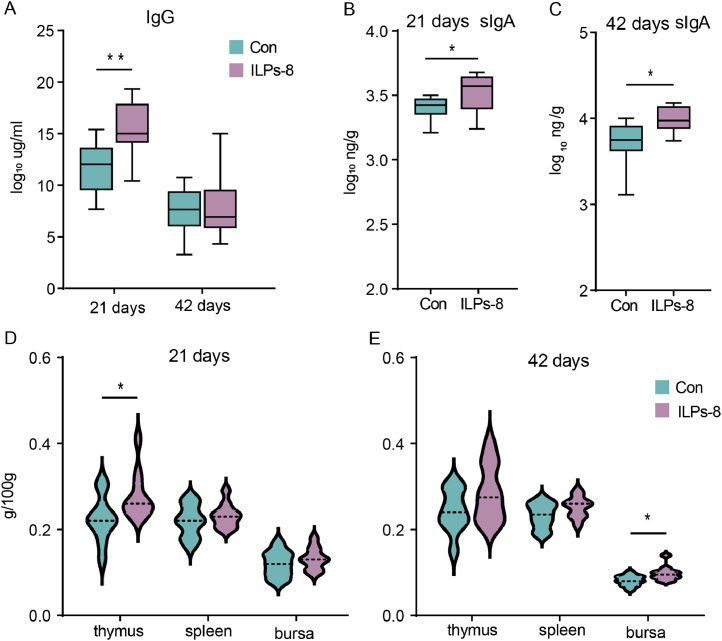
Changes in (A) immune organ indices, (B) serum IgG, and (C) small intestinal sIgA levels in the ILPs-8 group and the Con group at 21 and 42 days. Significance was marked by * *P* < 0.05, ** *P<* 0.01.

To evaluate the effects of ILPs-8 supplementation on the immune function of broilers, the relative weights of immune organs, serum IgG levels, and small intestinal sIgA concentrations were measured at 21 and 42 d of age. Broilers receiving ILPs-8 exhibited significantly higher serum IgG concentrations on day 21 and elevated sIgA levels in the small intestine at both 21 and 42 days compared to the Con group (*P* < 0.05) (Fig. 3A-C). Moreover, dietary supplementation with ILPs-8 significantly enhanced the thymus index on day 21 and bursa index on day 42 compared to the Con group (*P* < 0.05) (Fig. 3D-E) . However, the indices for the other immune organs showed no significant differences between the two groups (*P* > 0.05). Collectively, these findings underscore the immunomodulatory potential of ILPs-8 supplementation in broiler chickens.

### ILPs-8 regulated gut microbiota composition in broilers

**Fig. 4:**
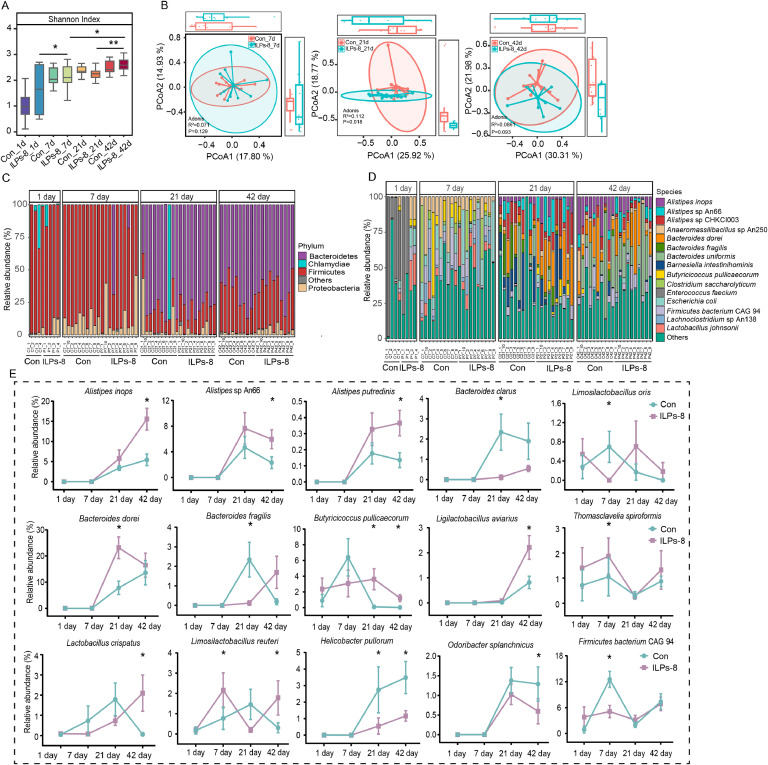
Diversity, species characteristics, and functional metabolic pathways of the cecal microbiota of broilers. (A) Shannon diversity index. (B) Bray-Curtis distance-based species-level PCoA score plots of the cecal microbiota at different time points. Distribution of cecal microbiota at (C) the phylum and (D) specie levels. (E) Species with significant differences between the ILPs-8 and Con groups at different time points. Significance was marked by * *P* < 0.05.

To elucidate the impact of ILPs-8 supplementation on gut microbiota composition, cecal microbial communities were assessed for alpha diversity (Shannon index) and beta diversity (PCoA and Adonis test). Although no significant difference in alpha diversity was observed between the ILPs-8 and Con groups at individual time points (*P* > 0.05; Fig. 4A), the Shannon index significantly increased with age in the ILPs-8 group, demonstrating progressive increments on days 7, 21, and 42 compared to preceding sampling points (*P* < 0.05). This age-associated shift was not observed in the Con group. Significant differences in microbiota structure between the ILPs-8 and Con groups were only observed on day 21 (R^2^ = 0.112, *P* = 0.018; Fig. 4B), suggesting that ILPs-8 supplementation markedly modulated the gut microbiota composition at this stage.

Further taxonomic analyses at the phylum and species levels revealed distinct microbial community dynamics associated with ILPs-8 supplementation. At the phylum, Bacteroidetes, Firmicutes, and Proteobacteria constituted over 95 % of the total cecal microbiota in both groups (Fig. 4C). At the species level, 15 species differed significantly between the Con and ILPs-8 groups (Fig. 4E). Specifically, on day 7, *Thomasclavelia spiroformis* and *Limosilactobacillus reuteri* relative abundance was elevated, whereas *Limosilactobacillus oris* and *Firmicutes bacterium* CAG 94 relative abundance was reduced in the ILPs-8 group (*P* < 0. 05). On day 21, the relative abundance of *Bacteroides dorei* and *Butyricicoccus pullicaecorum* was significantly increased, whereas the relative abundance of *Bacteroides clarus, Bacteroides fragilis*, and *Helicobacter pullorum* was significantly decreased in the ILPs-8 group compared to that in the Con group (*P* < 0. 05). On day 42, the relative abundance of *Alistipes inops, Alistipes* sp An66, *Ligilactobacillus aviarius, Alistipes putredinis, Butyricicoccus pullicaecorum, Limosilactobacillus reuteri*, and *Lactobacillus crispatus* was significantly greater, whereas those of *Helicobacter pullorum* and *Odoribacter splanchnicus* was significantly lower in the ILPs-8 group than in the Con group (*P* < 0. 05). These findings indicate a clear influence of ILPs-8 on the species-specific composition of the cecal microbiota of broiler chickens.

**Fig. 5:**
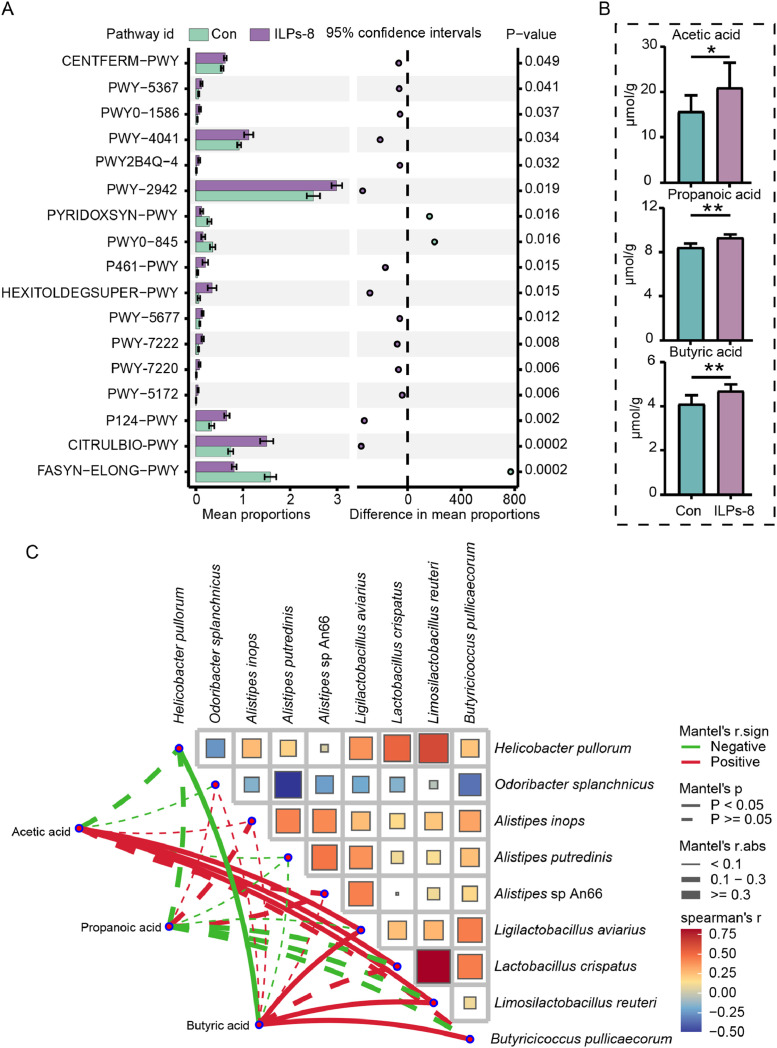
Functional metabolic pathways, short-chain fatty acid concentrations and correlation analysis of differential strains with short-chain fatty acids in the cecal microbiota of broilers on day 42. (A) Functional metabolic pathways of cecum microbiota significantly different between the ILPs-8 and Con groups. (B) Short-chain fatty acid concentrations in broiler cecal contents. (C) Correlation analysis of differential bacterial species with short-chain fatty acids. Significant were marked by * *P* < 0.05, ** *P<* 0.01.

Metagenomic functional profiling using the HUManN2 pipeline on day 42 identified 250 annotated metabolic pathways, of which 17 differed significantly between the two groups (*P* < 0.05) (Fig. 5A, Table S2). Notably, 14 pathways (e.g., pyruvate fermentation to butyric acid, L-citrulline and L-lysine biosynthesis, and succinate fermentation to butyric acid) were enriched in the ILPs-8 group, whereas three pathways (pyridoxal 5′-phosphate biosynthesis I, pyridoxal 5′-phosphate biosynthesis and salvage, and saturated fatty acid elongation) were predominantly enriched in the Con group (*P* < 0.05). These observations imply that ILPs-8 supplementation promotes distinct functional capacities in the gut microbiome. The differential enrichment of these microbially encoded functional pathways may contribute to the differences between the two groups.

Considering the enrichment of microbial genes involved in short-chain fatty acid (SCFA) metabolism, the concentrations of acetic, propionic, and butyric acids in the cecal contents were determined. Broilers in the ILPs-8 group exhibited significantly higher concentrations of these SCFAs than those in the Con group (*P* < 0.05) (Fig. 5B). Correlation analysis further identified significant associations between specific microbial species and SCFA production (Fig. 5C). Acetic acid was positively correlated with *Ligilactobacillus aviarius, Lactobacillus crispatus*, and *Limosilactobacillus reuteri*; butyric acid was positively correlated with *Ligilactobacillus aviarius, Limosilactobacillus reuteri*, and *Butyricoccus pullicaecorum*; and *Helicobacter pullorum* was significantly negatively correlated with butyric acid levels (*P* < 0.05). These correlations suggest that specific gut microbial taxa modulated by ILPs-8 may actively contribute to the metabolism of SCFAs, potentially playing a crucial role in maintaining broiler health.

### ILPs-8 modulated serum metabolites in broilers

**Fig. 6:**
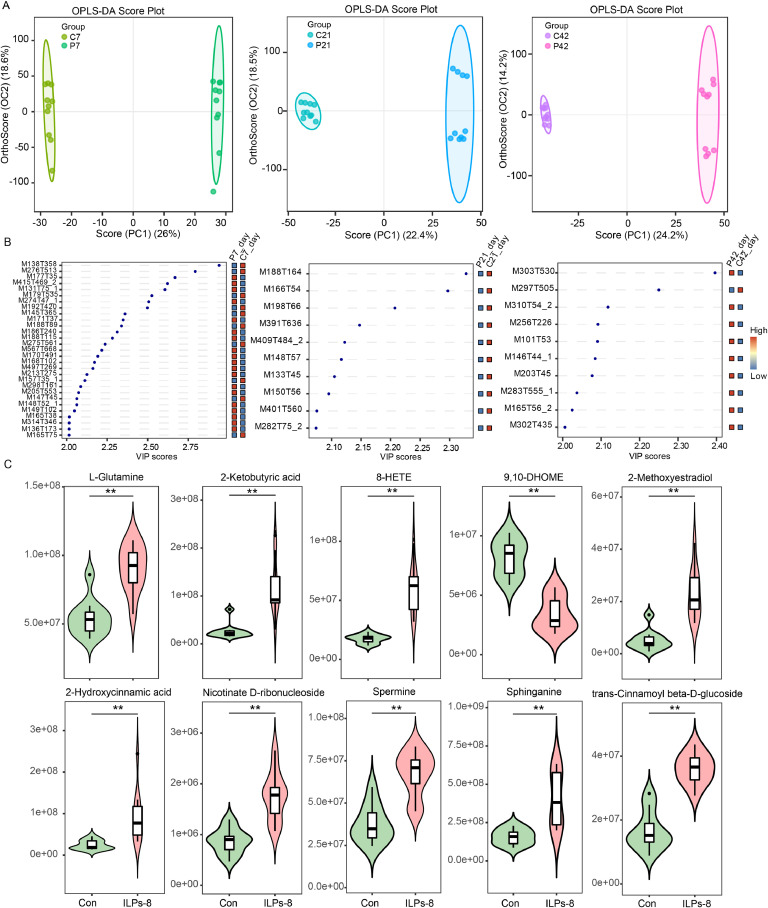
Comparison of serum metabolites at different time points in the control (Con) and ILPs-8 (ILPs-8) groups (*n* = 10). (A) Plot of OPLS-DA scores. (B) Metabolites that were significantly different were identified by comparing the two groups at the same time. The heat map on the right shows the relative abundance of each metabolite. (C) Relative amounts of significantly different metabolites at the end of the experiment. *P*-values (t-test) for comparisons between groups are shown. Significance was marked by ** *P<* 0.01.

To elucidate the physiological responses elicited by ILPs-8 supplementation in broilers, we analyzed the alterations in the serum metabolome at three critical developmental stages (7, 21, and 42 days of age). Score plots were visualized using OPLS-DA to demonstrate the differences in serum metabolites between the Con and ILPs-8 groups on different days (Fig. 6A). The resulting OPLS-DA models demonstrated strong predictive performance, with R^2^Y values of 0.999, 0.996, and 0.999 and Q^2^ values of 0.628, 0.649, and 0.806 at 7, 21, and 42 days, respectively. The Q^2^ values were all greater than 0.5, confirming the robustness and high predictive accuracy of these models. The clear separation between groups in the score plots further validated the effectiveness of OPLS-DA in distinguishing metabolite variations between the two groups (Fig. 6A). Using a stringent threshold (VIP score > 2, *P* < 0.05) in the hybrid metabolomic model, we identified 29, 10, and 10 significantly altered metabolite features on days 7, 21, and 42, respectively (Fig. 6B, Table S3). Notably, specific metabolites were consistently enriched in the ILPs-8 group compared to the Con group at each time point. At day 7, the prominent metabolites included 3-Hydroxyanthranilic acid, L-Glutamic acid, and L-Isoleucine (Fig. 6B, Table S3). At day 21, notable increases were observed in Racemethionine, Myriocin, and Deoxycholic acid levels (Fig. 6B, Table S3). At day 42, metabolites such as L-Glutamine, 2-Hydroxycinnamic acid, 2-Ketobutyric acid, and Spermine were significantly elevated in ILPs-8 supplemented broilers (Fig. 6C). Collectively, these metabolomic changes provide compelling evidence supporting the distinct physiological modifications associated with ILPs-8 supplementation in broiler chickens.

### Correlations between cecal microbiota, serum metabolites and phenotypic characteristics

**Fig. 7:**
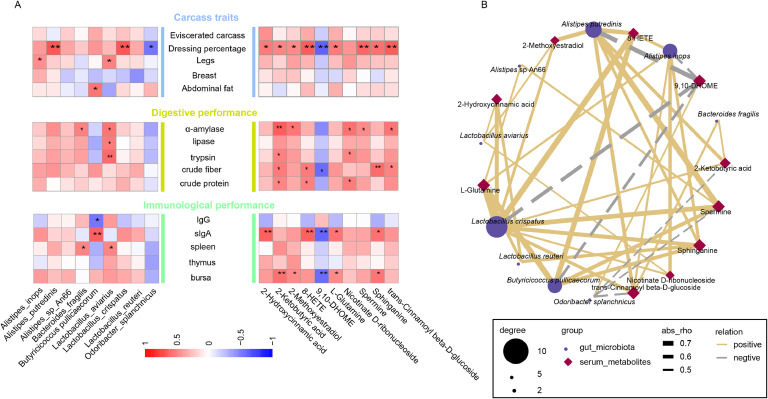
Correlation analysis between gut microbiota, serum metabolites, and phenotypes. (A) Correlation between gut microbiota, serum metabolites, and different phenotypic traits. (B) Correlation between the gut microbiota and differential serum metabolites.

Spearman correlation analyses were performed to investigate the associations between differential gut microbial species, serum metabolites, and phenotypic traits, including carcass characteristics, digestive efficiency, and immune function (Fig. 7A). *Alistipes putredinis* and *Lactobacillus crispatus* were positively correlated with dressing percentage (*P* < 0.05), whereas *Alistipes inops* and *Ligilactobacillus aviaries* were positively correlated with legs weight (*P* < 0.05). In addition, the relative abundance of *Butyricicoccus pullicaecorum* was positively correlated with abdominal fat (*P* < 0.05). Digestive enzyme activity was significantly and positively associated with *Ligilactobacillus aviarius* (*P* < 0.05). Immune parameters showed distinct microbial correlations; sIgA concentrations in the small intestine correlated positively with *Butyricicoccus pullicaecorum* (*P* < 0.05), whereas the spleen index correlated positively with both *Bacteroides fragilis* and *Ligilactobacillus aviarius* (*P* < 0.05). Conversely, the dressing percentage was significantly negatively correlated with *Odoribacter splanchnicus* (*P* < 0.05), and serum IgG levels were negatively associated with *Butyricicoccus pullicaecorum* (*P* < 0.05). For serum metabolites, most metabolites, except Nicotinate D-ribonucleoside, were positively correlated with dressing percentage (*P* < 0.05), whereas 9,10-DHOME showed a significant negative correlation (*P* < 0.05). Five metabolites, namely 2-Ketobutyric acid, 2-Methoxyestradiol, Nicotinate D-ribonucleoside, Spermine and trans-Cinnamoyl beta-D-glucoside- significantly positively correlated with α-amylase activity (*P* < 0.05). Both 2-Ketobutyric acid and Nicotinate D-ribonucleoside exhibited positive correlations with trypsin activity. Furthermore, four metabolites, including 2-Hydroxycinnamic acid, 8-HETE, L-Glutamine, and Sphinganine, were positively correlated with small intestinal sIgA levels (*P* < 0.05), whereas 9,10-DHOME was negatively correlated with sIgA levels (*P* < 0.05). Additionally, the bursa index was significantly positively correlated with 2-Ketobutyric acid, 2-Methoxyestradiol, L-Glutamine, and Sphinganine, but negatively correlated with 9,10-DHOME (*P* < 0.05).

Correlation network analysis further elucidated the interactions between cecal microbiota and serum metabolites (Fig. 7B). *Alistipes putredinis* emerged as a key microbial node, exhibiting significant positive correlations with 8-HETE, Sphinganine, Spermine, and 9,10-DHOME (*P* < 0.05). *Lactobacillus crispatus* also played a central role, with strong positive associations identified with l-Glutamine, 2-Hydroxycinnamic acid, and *Butyricicoccus pullicaecorum* (*P* < 0.05). Four microbial species, *Lactobacillus crispatus, Alistipes inops, Butyricicoccus pullicaecorum*, and *Limosilactobacillus reuteri,* showed significant positive correlations with L-Glutamine (*P* < 0.05). Conversely, *Odoribacter splanchnicus* abundance was negatively correlated with 2-Ketobutyric acid and spermine (*P* < 0.05), and 9,10-DHOME was negatively correlated with *Lactobacillus crispatus* and *Butyricoccus pullicaecorum* (*P* < 0.05). Collectively, these findings suggest that dietary supplementation with ILPs-8 may enhance growth performance, digestibility, and immune function in broilers by modulating cecal microbiota composition and serum metabolite profiles.

## Discussion

Under intensive poultry rearing conditions, the growth and development of broilers are frequently compromised by various stress factors, including heat stress, nutritional deficiencies, and overcrowding (Selvam, et al., 2017). The prolonged and indiscriminate use of antibiotics in poultry production has raised significant concerns due to their potential adverse effects on agricultural produce, livestock products, and public health (Abreu, et al., 2023). Consequently, numerous countries have implemented restrictions or outright bans on the use of antibiotics in poultry farming. Inactivated probiotics offer significant advantages in safety (eliminating risks of bacterial flora displacement and precluding transmission of antibiotic resistance genes), environmental stability (exhibiting tolerance to high temperatures, gastric acid, and bile), and storage stability (enabling extended shelf-life), making them particularly well-suited for feed processing applications. (Chen, et al., 2024; Jia, et al., 2025). Emerging evidence indicates that inactivated probiotics exhibit potent immunomodulatory, anti-inflammatory, and health-promoting properties (Incharoen, et al., 2019; Jin, et al., 2020; Zhu, et al., 2020), suggesting their potential to promote the growth and development of broilers and regulate their immune function. Against this backdrop, the present study was designed to investigate the effects of dietary supplementation with ILPs-8 on various biological parameters in broiler chickens. Additionally, a comprehensive multi-omics approach was used to identify alterations in health-associated parameters following ILPs-8 supplementation.

The current study demonstrated that dietary inclusion of ILPs-8 significantly enhanced the ADG of broilers during both the initial (days 1-21) and subsequent (days 22-42) growth phases. Additionally, a notable improvement in FCR was observed on days 1-21 and throughout the entire growth period (days 1-42). These findings align with previous reports on the beneficial effects of inactivated probiotics on broiler growth performance. For instance, Inchaccroen et al. (Incharoen, Charoensook, Onoda, Tatrakoon, Numthuam and Pechkong, 2019) reported enhanced growth performance following dietary supplementation with heat-treated *Lactiplantibacillus plantarum*l-137. Similarly, Khonyoung and Yamauchi (Khonyoung and Yamauchi, 2019) observed improved ADG and FCR in broilers receiving heat-treated Lactobacillus sakeensis HS-1. Digestive enzymes, such as trypsin, lipase, and amylase, play critical roles in the breakdown of proteins, lipids, and carbohydrates in the small intestine (Keomanivong, et al., 2017). In this study, broilers supplemented with ILPs-8 exhibited significantly increased α-amylase and trypsin activities in the small intestine compared to the Con group. This enhancement in enzymatic activity can likely be attributed to specific bioactive compounds within ILPs-8, which may stimulate endogenous enzyme synthesis and facilitate nutrient digestion. Morphological development of the small intestine, particularly the mucosal architecture, is a strong indicator of gastrointestinal health. Upon ingestion and enzymatic digestion of dietary components in the small intestine, nutrients are absorbed into the bloodstream via the intestinal mucosa and epithelial cell layers. This intricate process is pivotal in regulating nutrient transport and its subsequent metabolic utilization (Kiela and Ghishan, 2016). The findings indicate that ILPs-8 supplementation markedly increased VH in the duodenum and VCR in both the duodenum and ileum. Enhanced villus height effectively expands the intestinal absorptive surface area, thereby improving nutrient absorption capacity (Yaqoob, et al., 2022). Furthermore, improved mucosal morphology contributes significantly to epithelial cell integrity and maturation, which collectively optimize digestion efficiency and nutrient uptake (Gehart and Clevers, 2019; Shan, et al., 2012). Collectively, these results underscore the positive effects of dietary ILPs-8 supplementation on digestive enzyme activity and intestinal mucosal morphology, facilitating improved nutrient digestion, absorption, and overall metabolic efficiency. Ultimately, these physiological improvements translate into enhanced growth performance in broilers, underscoring the potential of ILPs-8 as a viable alternative to antibiotics in poultry production.

Maintaining optimal immune activity is essential for ensuring the healthy growth of broilers on poultry farms (Zhang, et al., 2023). Immunoglobulin G (IgG), constituting more than 75 % of serum antibodies, represents the predominant antibody class within the serum (Gondim, et al., 2021). Secretory immunoglobulin A (sIgA), primarily produced by lymphoid tissues located within the lamina propria of the respiratory and gastrointestinal mucous membranes, forms a critical immunological barrier on the mucosal surfaces (Zhen, et al., 2022). This protective barrier effectively prevents pathogenic adherence to the cell surface, thereby significantly mitigating the risk of infection and pathogenic invasion. The immune competence of poultry is closely associated with the absolute and relative masses of immune organs (Zhang, et al., 2021). The thymus, a key central immune organ, is instrumental in the differentiation and maturation of T lymphocytes (Gao, et al., 2017b). Similarly, the bursa of the falciparum, unique to avian species, functions as a central hub for B lymphocyte development, which is critical for humoral immunity (Cubas-Gaona, et al., 2021). Notably, the increased mass of immune organs following ILPs-8 administration, such as the thymus and bursa, provides compelling evidence of its role in enhancing immune organ development. In the present study, ILPs-8 supplementation significantly elevated the thymus index on day 21 and bursa index on day 42 compared to the control group. These observations underscore the potential efficacy of ILPs-8 in facilitating the maturation and proliferation of T- and B-lymphocytes. Collectively, these findings indicate that supplementation with ILPs-8 notably improves immune function in broilers, as demonstrated by increased immune organ weights and enhanced secretion of immune factors. Consequently, ILPs-8 has emerged as a promising agent for improving disease resistance and reducing stress in poultry production systems.

Early development is a critical period for animal growth, during which the microecological status of the gut significantly influences the health, growth trajectories, and physiological maturation of broilers. High gut microbiota diversity is typically positively correlated with improved health (Wosinska, et al., 2019). Consistent with prior findings, our study observed an initial increase in cecal microbial diversity during the first 7day post-hatch, which subsequently stabilized, indicating a rapid microbial colonization phase occurring early in broiler development (Feng, et al., 2023). Significant differences in microbial community composition were detected between the ILPs-8 supplemented group and the Con group at various developmental stages. Specifically, *Limosilactobacillus reuteri, Bacteroides dorei, Butyricicoccus pullicaecorum, Alistipes putredinis, Lactobacillus crispatus,* among other taxa, were notably enriched in the ILPs-8 group. Conversely, *Limosilactobacillus oris, Bacteroides fragilis, Helicobacter pullorum*, and *Odoribacter splanchnicus* were more abundant in the control group. *Limosilactobacillus reuteri* is recognized for its glucose fermentation capability, which yields metabolites such as lactic acid and ethanol. Moreover, supplementation with *Butyricoccus pullicaecorum* and *Alistipes inops* promotes the production of butyrate, acetate, propionate, and succinate. These metabolites have well-documented anti-inflammatory properties, reduce the abundance of pathogenic intestinal bacteria, and are critical for maintaining intestinal integrity, consequently lowering the incidence of intestinal lesions (Eeckhaut, et al., 2016). Thus, the elevated presence of these beneficial species may facilitate the early establishment of robust digestive and immune systems in broilers, enhancing their growth rates and feed-utilization efficiency. Furthermore, ILPs-8 supplementation notably reduced the abundance of *Helicobacter pullorum* throughout the experimental period, particularly during the later growth stages. *Helicobacter pullorum* predominantly colonizes the gastrointestinal tract of poultry, causing chronic inflammation, mucosal injury, and potentially severe conditions such as intestinal hemorrhage (Elrais, et al., 2022), negatively affecting growth and immunity (Fan, et al., 2022). The addition of ILPs-8 was also associated with an increased abundance of lactic acid bacteria in the cecum. This phenomenon is likely attributable to the organic acids, particularly lactic acid, produced by inactivated *Lactiplantibacillus plantarum*. These acids effectively lower intestinal pH, creating an environment that inhibits pathogenic bacteria while facilitating the proliferation of acid-tolerant beneficial microbes, such as *Lactobacillus* spp (Gao, et al., 2024). Thus, ILPs-8 supplementation fosters a favorable intestinal microbial ecosystem that promotes gastrointestinal health (Garbacz, 2022; Huang, et al., 2022; Li, et al., 2022). The changes observed in the gut microbiota composition were further corroborated by alterations in microbial metabolic functions. Specifically, pathways involved in the production of beneficial metabolites, such as butyric acid, lactic acid, acetic acid, lysine, and citrulline, were significantly enriched in the ILPs-8 group at the end of the experiment. SCFAs, notably butyrate and acetate, serve as vital energy substrates for intestinal epithelial cells, strengthen intestinal barrier function, and exert anti-inflammatory effects, thereby attenuating systemic inflammation (Ma, et al., 2022; Zou, et al., 2021). Interestingly, the metabolic pathways responsible for pyridoxal 5′-phosphate synthesis and fatty acid biosynthesis were predominantly enriched in the control group. Pyridoxal 5′-phosphate biosynthesis pathways have established roles in modulating host immune responses and inflammation, influencing gut microbiota composition and function, either directly or indirectly (Agarwal and Allamaneni, 2004).

The gut microbiota plays a crucial role in modulating the host’s serum metabolic status (Wang, et al., 2020). At the conclusion of the experiment, significant alterations in serum metabolites were observed in broilers treated with ILPs-8, marked by elevated levels of l-Glutamine, 8-HETE, 2-Methoxyestradiol, 2-Hydroxycinnamic acid, Spermine, and Sphinganine and reduced levels of 2-ketobutyric acid. These differential serum metabolites are intricately related to physiological processes, including energy metabolism and immune modulation. The increased concentration of l-Glutamine observed in the ILPs-8 treatment group may have resulted from microbiota-driven alterations. ILPs-8 appears to enhance l-Glutamine synthesis and metabolism by improving intestinal health and facilitating nutrient absorption, consequently augmenting energy supply and immune function (He, et al., 2022; Shah, et al., 2020). Conversely, a reduction in 2-Ketobutyric acid, a metabolite strongly associated with amino acid and glucose metabolism, suggests gut microbime-mediated regulation of energy metabolism pathways, likely through modulation of amino acid and glucose homeostasis (Chen, et al., 2023; Hao, et al., 2024). Moreover, the elevated levels of 8-HETE, a metabolite commonly associated with inflammatory and immune responses, imply that ILPs-8 may bolster host resistance to disease by modulating immune responses and enhancing gut barrier integrity, consistent with the findings of previous studies (Cui, et al., 2023; Zhang, et al., 2018). Additionally, the significant increase in 2-Hydroxycinnamic acid, a compound known for its antioxidant and anti-inflammatory properties (Sharma and Singh, 2012), further indicates that ILPs-8 may enhance the overall health status of broilers by strengthening antioxidant defenses and mitigating oxidative stress. Furthermore, the observed increase in spermine levels in the ILPs-8 group aligns with existing evidence that spermine promotes immune cell proliferation and activity, thereby amplifying immune responses and improving resistance to infections and diseases (Xu, et al., 2024). These findings collectively highlight the potential role of ILPs-8 in reinforcing the intestinal barrier and optimizing immune function.

Overall, our results underscore that ILPs-8 administration significantly enhances broiler growth performance and health, as demonstrated by improved small intestine development, increased digestive enzyme activity, and beneficial modifications to both cecal microbiota and serum metabolic profiles. Importantly, employing inactivated probiotics (ILPs-8) represents a highly promising intervention strategy, circumventing the issues associated with probiotic viability and avoiding the physiological stress or rejection typically encountered in neonatal broilers subjected to direct probiotic supplementation via diet or by gavage administration.

## Conclusion

In conclusion, this study demonstrated that dietary supplementation with ILPs-8 significantly enhanced the growth performance and immune response of broiler chickens. These beneficial outcomes appear to be mediated through several interconnected mechanisms, including elevated activity of small intestinal digestive enzymes, improved intestinal morphology, enriched colonization of beneficial cecal bacteria coupled with reduced prevalence of pathogenic bacteria, and serum metabolite modulation. These findings provide valuable insights into the functional advantages of inactivated probiotics, with practical implications for optimizing productivity in intensive poultry farming settings. Consequently, this study advances our current understanding and broadens the potential industrial applications of inactivated probiotics.

## CRediT authorship contribution statement

**Yangbo Jiao:** Writing – original draft, Formal analysis, Visualization. **Weiqiang Huang:** Methodology. **Qihang Zhang:** Data curation. **Lin Liu:** Visualization. **Jie Zhao:** Funding acquisition, Project administration, Writing – review & editing. **Yongfu Chen:** Funding acquisition, Writing – review & editing, Supervision.

## Disclosures

The authors declare that they have no known competing financial interests or personal relationships that could have appeared to influence the work reported in this paper.

## Appendix Supplementary materials

Image, application 1
