# Supplementary material for: Inactivated Lactiplantibacillus plantarum Ps-8 enhances growth performance and intestinal health in broiler chickens via gut microbiota and serum metabolite modulation
Source: Poult Sci. 2025 Jul 26;104(10):105611. doi: 10.1016/j.psj.2025.105611 (PMC12341604; doi:10.1016/j.psj.2025.105611)
Supplement: Supplementary file 1 [file mmc1.docx]

**Inactivated *Lactiplantibacillus plantarum* Ps-8 enhances growth performance and intestinal health in broiler chickens via gut microbiota and serum metabolite modulation**

Yangbo Jiao ^1,2,3^, Weiqiang Huang ^4^, Qihang Zhang ^1,2,3^, Lin Liu ^1,2,3^, Jie Zhao ^1,2,3,*^, Yongfu Chen^1,2,3,*^

* Corresponding authors:

Jie Zhao, E-mail address: nmgzj@imau.edu.cn (J. Zhao);

Yongfu Chen, E-mail address: [nmgyfchen@126.com](mailto:nmgyfchen@126.com) (Y. Chen)

^1^ Key Laboratory of Dairy Biotechnology and Engineering, Ministry of Education, Inner Mongolia Agricultural University, Hohhot 010018, China

^2^ Key Laboratory of Dairy Products Processing, Ministry of Agriculture and Rural Affairs, Inner Mongolia Agricultural University, Hohhot 010018, China

^3^ Inner Mongolia Key Laboratory of Dairy Biotechnology and Engineering, Inner Mongolia Agricultural University, Hohhot 010018, China

^4^ Inner Mongolia SCITOP Microecological Technology Development Co., Ltd., Hohhot 010018, China

Table S1 Composition of ILPs-8 bacterial powder.

|  | Metabolites | Signal intensity |
| --- | --- | --- |
| 1 | Isoquinoline | 27998926.35 ± 629086.64 |
| 2 | Miltefosine | 23095356.54 ± 167846.88 |
| 3 | L-Phenylalanine | 19027134.4 ± 451296.97 |
| 4 | L-Arginine | 7708733.5 ± 1193965.87 |
| 5 | Stearidonic acid | 4252687.35 ± 117275.13 |
| 6 | Cauloside C | 3495674.41 ± 139291.81 |
| 7 | Adenine | 123667.49 ± 8527.34 |
| 8 | 16-Hydroxyhexadecanoic acid | 2897911.34 ± 104189.4 |
| 9 | Oxalic acid | 2105320.67 ± 78533.86 |
| 10 | L-Lysine | 51598.79 ± 4598.05 |
| 11 | Acetylcholine | 1115091.03 ± 36310.27 |
| 12 | 2-Hydroxycinnamic acid | 1069631.35 ± 98181.52 |
| 13 | Guanine | 1613305 ± 13543.35 |
| 14 | Cytosine | 360419.78 ± 6608.04 |
| 15 | L-Proline | 509282.61 ± 183397.36 |
| 16 | Fucoxanthin | 19531.8 ± 2024.31 |
| 17 | Spermine | 276371.86 ± 42020.94 |
| 18 | Simvastatin | 237892.5 ± 7727.76 |
| 19 | L-Ornithine | 226114.2 ± 5885.38 |
| 20 | Succinic acid | 174400.57 ± 7114.33 |
| 21 | Damsin | 17069.51 ± 138.79 |
| 22 | 1H-Indole-3-propanoic acid | 124813.5 ± 35213.17 |
| 23 | esculetin | 119652.88 ± 120840.51 |
| 24 | Aspartyl adenylate | 14955.45 ± 1338.9 |
| 25 | 4-Hydroxycinnamic acid | 111205.78 ± 35648.33 |
| 26 | 5-Methoxyindole-3-acetic acid | 79764.05 ± 9392.58 |
| 27 | Vitamin K | 67721.74 ± 18114.07 |
| 28 | (+)-Isocorydine | 64290.65 ± 3350.12 |
| 29 | Carnosic acid | 59401.92 ± 14601.77 |
| 30 | Linolenic acid | 58098.83 ± 3503.11 |
| 31 | Boldine | 54754.82 ± 5525.5 |
| 32 | L-Asparagine | 53392.27 ± 7324.53 |
| 33 | Discodermolide | 49786.82 ± 2260.04 |
| 34 | falcarindiol | 46756.8 ± 3234.53 |
| 35 | Histamine | 0 ± 1569.85 |
| 36 | Oxymatrine | 36194.38 ± 4455.58 |
| 37 | 4-Guanidinobutanamide | 23102.68 ± 3658.11 |
| 38 | Dehydrocostus lactone | 20400 ± 5025.46 |
| 39 | Biotinyl-CoA | 19928.77 ± 423.08 |
| 40 | Hordatine A | 13944.91 ± 253.71 |
| 41 | gamma-Oryzanol | 14978.1 ± 623.8 |
| 42 | Squalamine | 14996.25 ± 2447.05 |

Table S2 The name and classification of different pathways.

| Pathway id | Name of the pathway | Classification |
| --- | --- | --- |
| CENTFERM-PWY | Pyruvate fermentation to butanoate | Generation of Precursor Metabolites and Energy → Fermentation → Fermentation of Pyruvate; |
|  |  | Generation of Precursor Metabolites and Energy → Fermentation→  Fermentation to Short-Chain Fatty Acids → Fermentation to Butanoate; |
| PWY-5367 | petroselinate biosynthesis | Biosynthesis → Fatty Acid and Lipid Biosynthesis → Fatty Acid Biosynthesis →  Unsaturated Fatty Acid Biosynthesis |
| PWY-4041 | γ-glutamyl cycle | Biosynthesis → Other Biosynthesis → Reductant Biosynthesis |
| PWY0-1586 | Peptidoglycan maturation (meso-diaminopimelate containing) | Biosynthesis → Cell Structure Biosynthesis → Cell Wall Biosynthesis → Peptidoglycan Biosynthesis |
| PWY2B4Q-4 | Lipid IVA biosynthesis | Biosynthesis → Cell Structure Biosynthesis → Lipopolysaccharide Biosynthesis → Lipid IVA Biosynthesis; |
|  |  | Biosynthesis → Fatty Acid and Lipid Biosynthesis → Lipid IVA Biosynthesis; |
|  |  | Glycan Pathways → Lipopolysaccharide Biosynthesis → Lipid IVA Biosynthesis |
| PWY-2942 | L-lysine biosynthesis III | Biosynthesis → Amino Acid Biosynthesis → Proteinogenic Amino Acid Biosynthesis →  L-lysine Biosynthesis |
| PYRIDOXSYN-PWY | Pyridoxal 5'-phosphate biosynthesis I | Biosynthesis → Cofactor, Carrier, and Vitamin Biosynthesis → Enzyme Cofactor Biosynthesis →  Vitamin B6 Biosynthesis; |
|  |  | Biosynthesis → Cofactor, Carrier, and Vitamin Biosynthesis → Vitamin Biosynthesis →  Vitamin B6 Biosynthesis |
| PWY0-845 | Superpathway of pyridoxal 5'-phosphate biosynthesis and salvage | Biosynthesis → Cofactor, Carrier, and Vitamin Biosynthesis → Enzyme Cofactor Biosynthesis →  Vitamin B6 Biosynthesis; |
|  |  | Biosynthesis → Cofactor, Carrier, and Vitamin Biosynthesis → Vitamin Biosynthesis →  Vitamin B6 Biosynthesis |
| P461-PWY | Hexitol fermentation to lactate, formate, ethanol and acetate | Degradation/Utilization/Assimilation → Alcohol Degradation → Alditol Degradation; |
|  |  | Degradation/Utilization/Assimilation → Carbohydrate Degradation → Alditol Degradation; |
|  |  | Generation of Precursor Metabolites and Energy → Fermentation → Fermentation of Pyruvate →  Pyruvate Fermentation to Ethanol; |
|  |  | Generation of Precursor Metabolites and Energy → Fermentation → Fermentation to Alcohols→  Pyruvate Fermentation to Ethanol; |
|  |  | Generation of Precursor Metabolites and Energy → Fermentation → Fermentation to  Short-Chain Fatty Acids → Fermentation to Acetate; |
|  |  | Generation of Precursor Metabolites and Energy → Fermentation → Fermentation to  Short-Chain Fatty Acids → Fermentation to Lactate |
| HEXITOLDEGSUPER-PWY | Superpathway of hexitol degradation (bacteria) | Degradation/Utilization/Assimilation → Alcohol Degradation → Alditol Degradation |
| PWY-5677 | Succinate fermentation to butanoate | Generation of Precursor Metabolites and Energy → Fermentation → Fermentation to  Short-Chain Fatty Acid →Fermentation to Butanoate |
| PWY-7222 | Guanosine deoxyribonucleotides de novo biosynthesis Ⅱ | Biosynthesis → Nucleoside and Nucleotide Biosynthesis→2'-Deoxyribonucleotide Biosynthesis →  Purine Deoxyribonucleotide De Novo;  Biosynthesis → Guanosine Deoxyribonucleotide De Novo Biosynthesis; |
|  |  | Biosynthesis → Nucleoside and Nucleotide Biosynthesis → Purine Nucleotide Biosynthesis →  Purine Nucleotide De Novo Biosynthesis → Purine Deoxyribonucleotide De Novo Biosynthesis →  Guanosine |
| PWY-7220 | Adenosine deoxyribonucleotides de novo biosynthesis II | Biosynthesis → Nucleoside and Nucleotide Biosynthesis → 2'-Deoxyribonucleotide Biosynthesis →  Purine Deoxyribonucleotide De Novo;  Biosynthesis → Adenosine Deoxyribonucleotide De Novo Biosynthesis; |
|  |  | Biosynthesis → Nucleoside and Nucleotide Biosynthesis → Purine Nucleotide Biosynthesis → Purine Nucleotide De Novo Biosynthesis → Purine Deoxyribonucleotide De Novo Biosynthesis → Adenosine Deoxyribonucleotide De Novo Biosynthesis |
| PWY-5172 | Superpathway of acetyl-CoA biosynthesis | Generation of Precursor Metabolites and Energy → Acetyl-CoA Biosynthesis |
| P124-PWY | Bifidobacterium shunt | Degradation/Utilization/Assimilation → Carbohydrate Degradation → Sugar Degradation; |
|  |  | Generation of Precursor Metabolites and Energy → Fermentation → Fermentation to  Short-Chain Fatty Acids → Fermentation to Acetate; |
|  |  | Generation of Precursor Metabolites and Energy → Fermentation → Fermentation to  Short-Chain Fatty Acids → Fermentation to Lactate |
| CITRULBIO-PWY | L-citrulline biosynthesis | Biosynthesis→ Amino Acid Biosynthesis → Other Amino Acid Biosynthesis → L-citrulline Biosynthesis |
| FASYN-ELONG-PWY | Fatty acid elongation – saturated | Biosynthesis → Fatty Acid and Lipid Biosynthesis → Fatty Acid Biosynthesis |

Table S3 Significantly different metabolites in serum at 7, 21 and 42 days of age.

|  | Compound name | Compound id | Mean_Con | Mean_ILPs-8 | *P*-Value | VIP |
| --- | --- | --- | --- | --- | --- | --- |
| Con_7day VS ILPs-8_7day | 3-Hydroxyanthranilic acid | M136T173 | 1940975.37 | 2442579.23 | 0.025405308 | 2.015304367 |
|  | 4-Oxoglutaramate | M145T365 | 144569827 | 113418896.2 | 0.008229238 | 2.359930498 |
|  | L-Lysine | M147T45 | 992201013.1 | 657268865.5 | 0.031175416 | 2.062600568 |
|  | L-Glutamic acid | M148T52_1 | 27101693.14 | 50567452.53 | 0.027703326 | 2.062166874 |
|  | (S)-2-Methylmalate | M149T102 | 77364999.19 | 81952372.69 | 0.028578251 | 2.048059736 |
|  | Uracil 5-carboxylate | M157T35_1 | 17674872.09 | 13461015.41 | 0.020913469 | 2.112463574 |
|  | 3,4-Dihydroxyphenylpropanoate | M165T75 | 704989285.6 | 561458631.7 | 0.030711461 | 2.014995536 |
|  | D-synephrine | M168T102 | 14210932.47 | 18194445.94 | 0.016040955 | 2.171844698 |
|  | Diphenylamine | M170T491 | 13573536.03 | 25008300.11 | 0.017187307 | 2.192238628 |
|  | Azelaic acid | M171T37 | 4319411.37 | 7278906.52 | 0.00799305 | 2.348739209 |
|  | N-Formyl-L-methionine | M177T35 | 20799683.93 | 35953769.54 | 0.001622549 | 2.668344162 |
|  | 2-Keto-6-acetamidocaproate | M188T89 | 268779393.4 | 112649488.8 | 0.010413948 | 2.338073853 |
|  | N,N-Diethyl-m-toluamide | M192T420 | 9468385.74 | 6929184.32 | 0.003779319 | 2.49821229 |
|  | Homatropine | M276T513 | 35020177.24 | 24325408.83 | 0.000874212 | 2.793278329 |
|  | 5'-Methylthioadenosine | M298T161 | 32604470.62 | 42875651.8 | 0.021454941 | 2.086725483 |
|  | Pergolide | M314T346 | 187903.81 | 675301.47 | 0.03530758 | 2.015616542 |
|  | Sodium deoxycholate | M415T469_2 | 153637950.2 | 298002007.2 | 0.002381755 | 2.622171087 |
|  | Telmisartan | M497T269 | 276810.13 | 545021.45 | 0.014895034 | 2.161904171 |
|  | Capsanthin | M567T668 | 16808716.92 | 25839400.08 | 0.013059749 | 2.216435615 |
|  | L-Isoleucine | M131T75_1 | 6283225.31 | 8882556.11 | 0.000160668 | 2.606325755 |
|  | 6-Hydroxynicotinic acid | M138T358 | 2626965.6 | 1302729.34 | 7.70514E-07 | 2.940541817 |
|  | Terephthalate | M165T38 | 777073.98 | 951833.16 | 0.006783091 | 2.016725122 |
|  | Theophylline | M179T535 | 509021420.4 | 150890739.8 | 0.001109526 | 2.525232784 |
|  | Pantothenol | M186T240 | 340316.68 | 598358.15 | 0.000525928 | 2.312057365 |
|  | Kynurenic acid | M188T115 | 2075506.33 | 3141903.03 | 0.002584707 | 2.282537441 |
|  | Methyl jasmonate | M205T553 | 3579722.48 | 7083906.59 | 0.004037066 | 2.074018595 |
|  | Deoxyribose 5-phosphate | M213T275 | 903307.39 | 2434310.25 | 0.006170864 | 2.125230281 |
|  | N2-gamma-Glutamylglutamine | M274T47_1 | 8415730.61 | 12795761.91 | 0.00034927 | 2.506008182 |
|  | Stearidonic acid | M275T561 | 32948827.09 | 20228505.96 | 0.001715103 | 2.237163891 |
| Con_21day VS ILPs-8_21day | Ornithine | M133T45 | 18957870.88 | 29167945.73 | 0.000293671 | 2.105035668 |
|  | 2-Hydroxyglutarate | M148T57 | 20340475.18 | 32766033.34 | 0.000301571 | 2.11662789 |
|  | Racemethionine | M150T56 | 98509378.63 | 137642725.6 | 0.000250191 | 2.095402748 |
|  | Levonordefrin | M166T54 | 3275154.76 | 5359821.98 | 2.93885E-05 | 2.296977781 |
|  | N-Alpha-acetyllysine | M188T164 | 40642285.27 | 74047072 | 1.64733E-05 | 2.327895583 |
|  | Myriocin | M401T560 | 3157819.21 | 5521151.01 | 0.000191441 | 2.075103863 |
|  | Procollagen 5-hydroxy-L-lysine | M198T66 | 2217001.01 | 2519196.61 | 0.000133109 | 2.207355509 |
|  | Guanosine | M282T75_2 | 2174345.42 | 3377542.46 | 0.000526507 | 2.07377307 |
|  | Deoxycholic acid | M391T636 | 20752560.58 | 33184274.69 | 0.000304583 | 2.147454771 |
|  | LysoPA(16_0_0_0) | M409T484_2 | 31595581.56 | 61217945.85 | 0.000240773 | 2.122313177 |
| Con_42day VS ILPs-8_42day | L-Glutamine | M146T44_1 | 54425751.05 | 90806024.56 | 4.12794E-05 | 2.084483975 |
|  | 2-Hydroxycinnamic acid | M165T56_2 | 24699033.88 | 93520978.44 | 7.61584E-05 | 2.024539616 |
|  | Spermine | M203T45 | 37383504.44 | 67409400.7 | 3.52326E-05 | 2.076521197 |
|  | Nicotinate D-ribonucleoside | M256T226 | 868545.06 | 1759127.37 | 3.57698E-05 | 2.091517924 |
|  | 9,10-DHOME | M297T505 | 8153355.49 | 3389602.96 | 2.47622E-05 | 2.249985231 |
|  | Sphinganine | M302T435 | 156787543.3 | 405660793.1 | 0.000123779 | 2.005652859 |
|  | 8-HETE | M303T530 | 17462755.21 | 59808801.47 | 4.67213E-08 | 2.396146909 |
|  | 2-Ketobutyric acid | M101T53 | 26473674.6 | 116361033.7 | 4.76275E-06 | 2.090476537 |
|  | 2-Methoxyestradiol | M283T555_1 | 5167351.41 | 22935527.82 | 1.23977E-05 | 2.03675098 |
|  | trans-Cinnamoyl beta-D-glucoside | M310T54_2 | 16567862.66 | 35997198.47 | 2.00689E-05 | 2.117682767 |
